# *Eclipse*: a Python package for alignment of two or more nontargeted LC-MS metabolomics datasets

**DOI:** 10.1093/bioinformatics/btaf290

**Published:** 2025-05-10

**Authors:** Daniel S Hitchcock, Jesse N Krejci, Chloe E Sturgeon, Courtney A Dennis, Sarah T Jeanfavre, Julian R Avila-Pacheco, Clary B Clish

**Affiliations:** Metabolomics Platform, Broad Institute of MIT and Harvard, Cambridge, MA 02142, United States; Metabolomics Platform, Broad Institute of MIT and Harvard, Cambridge, MA 02142, United States; Metabolomics Platform, Broad Institute of MIT and Harvard, Cambridge, MA 02142, United States; Metabolomics Platform, Broad Institute of MIT and Harvard, Cambridge, MA 02142, United States; Metabolomics Platform, Broad Institute of MIT and Harvard, Cambridge, MA 02142, United States; Metabolomics Platform, Broad Institute of MIT and Harvard, Cambridge, MA 02142, United States; Metabolomics Platform, Broad Institute of MIT and Harvard, Cambridge, MA 02142, United States

## Abstract

**Summary:**

Nontargeted LC-MS (liquid chromatography–tandem mass spectrometry) metabolomics datasets contain a wealth of information but present many challenges during analysis and processing. Often, two or more independently processed datasets must be aligned to form a complete dataset, but existing software does not fully meet our needs. For this, we have created an open-source Python package called *Eclipse*. *Eclipse* uses a novel graph-based approach to handle complex matching scenarios that arise from *n > 2* datasets.

**Availability and implementation:**

*Eclipse* is open source (https://github.com/broadinstitute/bmxp) and can be installed via “pip install bmxp.”

## 1 Introduction

Nontargeted liquid chromatography–tandem mass spectrometry (LC-MS) is a powerful methodology for inspecting the metabolic state of a biological specimen (Clish 2015). In a routine data processing workflow, feature extraction software converts raw instrument files to tabular datasets by identifying and integrating thousands of features. Each feature is reported with its chromatographic retention time (RT) and mass-to-charge ratio (*m/z*) (Smith *et al.* 2006, Pluskal *et al.* 2010). While many features receive chemical labels (annotations), a substantial portion remain unannotated. The unannotated space contains features of biological significance (Chen *et al.* 2022, Tahir *et al.* 2022, Vatanen *et al.* 2022), but presents challenges when attempting to concatenate datasets that have been acquired and processed separately, i.e. alignment (Smith *et al.* 2015). These challenges are exacerbated when *n > 2* datasets are introduced, leading to complex matching that cannot be fully represented in tabular data (Supplementary Fig. S1). While some solutions to align datasets based on feature descriptors exist (Brunius *et al.* 2016, Koch *et al.* 2016, Mak *et al.* 2020, Habra *et al.* 2021, 2024, Climaco Pinto *et al.* 2022), none satisfy all our requirements, specifically that it must run robustly in default settings, not produce multiple matches, be written in Python, and align *n > 2* datasets, with results being independent of dataset order.

For this, we developed *Eclipse* (https://github.com/broadinstitute/bmxp). *Eclipse* uses a novel graph-based alignment strategy which natively accommodates *n > 2* datasets. The output of *Eclipse* may be customized for a variety of experimental use cases, such as generating a combined dataset for processing or identifying overlapping features in disparate matrices.

## 2 *Eclipse* overview

**Figure 1.**
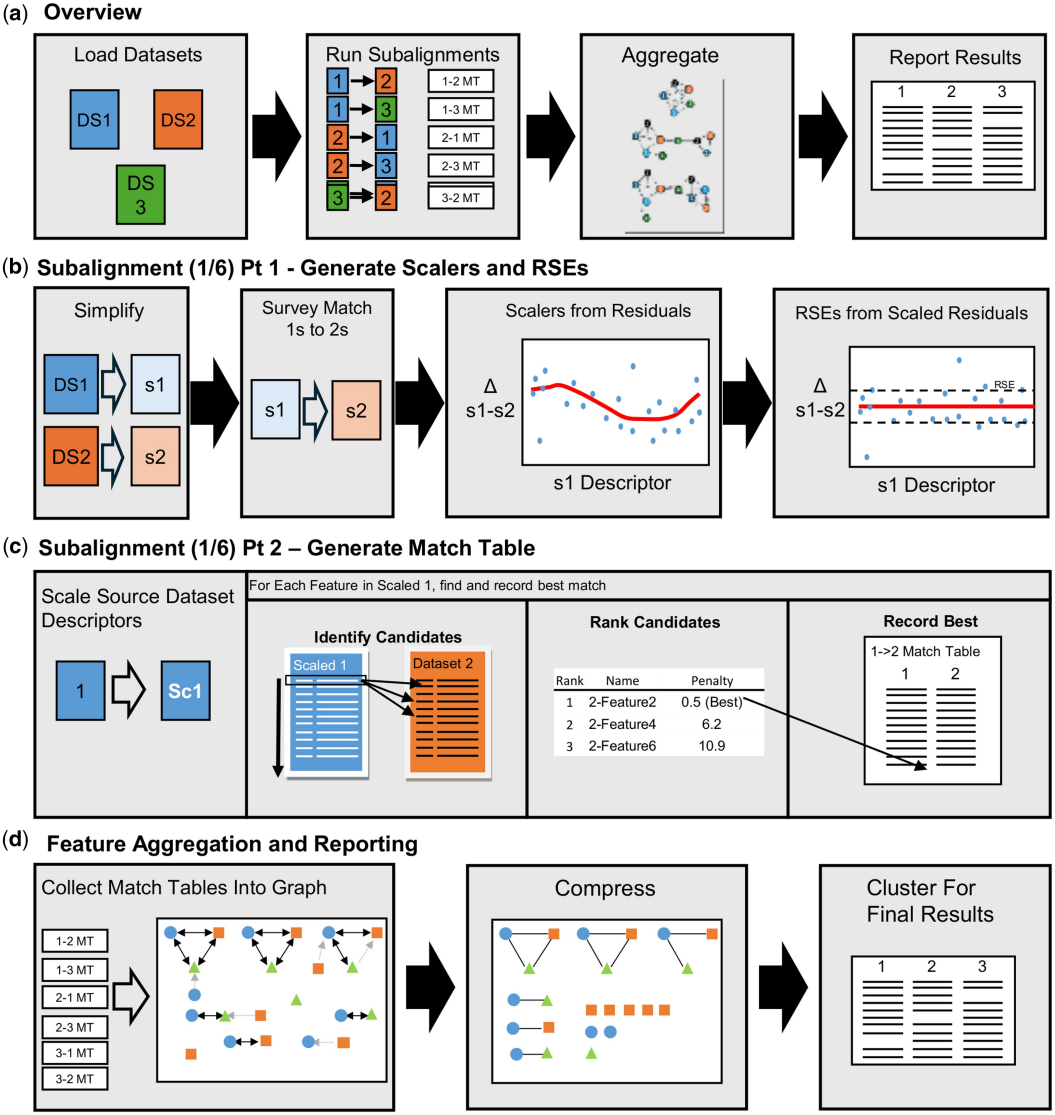
Overview of eclipse. (a) High level overview of the Eclipse algorithm with a three-dataset example. (b) Generation and scalers in the DS1→DS2 subalignment, one of six to be run. Datasets are simplified (s1, s2), then survey-matched. Scalers are generated from the residuals of each descriptor (RT, *m*/*z*, Intensity), then subtracted to reveal the residual square error. (c) Match table generation of the DS1*→*DS2 subalignment. DS1 is scaled (1→Sc1), then each feature is queried to DS2. DS2 that fall within ±6 RSEs of all descriptors are ranked, and the best match is recorded in the DS1→DS2 match table. (d) Aggregation and reporting of alignment results. Once all subalignments have been run, they are collected into a directed graph. The graph is compressed and clustered to produce a results table.

*Eclipse* aligns multiple datasets by first running directed alignments between dataset pairs (e.g. *DS1→DS2*, *DS2→DS1*), identifying corresponding features comparing the descriptors RT, *m/z*, and average Intensity (Fig. 1a) and producing subalignment match tables. We refer to these as subalignments. In each subalignment, one dataset acts as the *Source* (left of the arrow) and the other as the *Target* (right of the arrow). It is important to note that each subalignment is distinct, e.g. *DS1→DS2* is performed independently of the reverse, *DS2→DS1*. Next, these match tables are then combined in a graph, and finally tabular outputs can be generated via a customizable clustering algorithm.

### 2.1 Subalignment matching

Subalignment match tables are generated in two steps: (i) surveying the subalignment pair to determine inter-dataset scalers and scoring parameters (Fig. 1b), and (ii) recording best matches (Fig. 1c). Feature comparisons are performed in transformed spaces: RT by absolute differences (*linear*), *m/z* by *ppm*, and Intensity by *log10* scaling. *Eclipse* otherwise treats all descriptors identically, and they can be removed, or replaced.

First, each subalignment generates RT, *m/z*, and Intensity scalers to account for inter-dataset trends. *Source* and *Target datasets* are simplified by removing features within close proximity to neighbors (default thresholds: RT ± 0.5 min, *m/z* ± 15 ppm, Intensity ± 2 log10) (Supplementary Fig. S2). A survey match using these thresholds identifies *Source→Target* match pairs. Residuals from the matches are plotted against the *Source* descriptor values, and the resulting LOWESS fit defines the scalers for each descriptor. The scalers are applied to adjust the *Source* descriptor values to account for inter-dataset trends. After scaling, the remaining inter-dataset fluctuations (noise) are used to calculate the standard deviation of the residuals as the Residual Standard Error (RSE). RSE is used to weigh descriptor contributions and set cutoffs during matching.

Next, the *Source* dataset is scaled to the *Target* dataset, and potential matches are found by identifying *Target* features within ±6 RSE of the Source’s RT, *m/z*, and Intensity (Fig. 1c). Potential matches are ranked by a penalty score (Supplementary Equation S1), and the best-ranked match, along with its penalty, is recorded in the subalignment match table.

### 2.2 Feature aggregation and clustering

Once all subalignments have been completed, a Combined Dataset may be produced. All subalignment match tables are loaded into a directed graph (Fig. 1d), with features as nodes, matches as edges, and match penalties as edge weights. The graph is then converted to an undirected graph, retaining only bidirectional matches, with penalties from these edges summed. A clustering algorithm then ranks valid groups (described below), records the best group, removes it from the graph, and repeats until no valid groups remain (Supplementary Fig. S3a). This process eliminates redundant matches and ensures that dataset order does not influence the results. By default, valid groups must contain a member from all datasets and be fully interconnected (a clique), but these criteria can be customized. Users can specify the *minimum group size*, *minimum clique size*, and *diameter*, where *1* enforces strict cliques (“clique mode”), *2* allows one node in a clique to have neighbors, and *3* allows all nodes in a clique to have neighbors (Supplementary Fig. S3b). Groups are ranked by group size (higher is better), maximum clique size (higher is better), number of edges (higher is better), and total edge penalty (lower is better), as demonstrated in Supplementary Fig. S4.

## 3 Methods

Samples within LC-MS datasets (denoted as DS1 through DS11) were acquired on instruments comprised of Shimadzu Nexera X2 U-HPLCs coupled to Thermo Exactive series orbitrap mass spectrometers. DS1-4, DS10, and DS11 were created from pooled reference samples in distinctly processed human plasma datasets. DS5-9 were derived from datasets of various rodent tissues. All datasets were acquired using the same HILIC-pos method (Mascanfroni *et al.* 2015). Feature extraction was performed using Progenesis QI and features were annotated based on comparison to known LC-MS standards. Dataset information, including specific instrument models and acquisition dates, is summarized in Supplementary Table S1. All alignments were performed on an AMD Ryzen 5 3500x Windows 11 PC, running Python 3.12 and BMXP version 0.2.4 and Eclipse 0.2.3, using default settings unless otherwise noted. The benchmark times did not include file I/O. Data and scripts are available in the Supplementary Information. Spurious matches were reported as the total number of rows (matches) that contained an annotation mismatch.

## 4 Results and discussion

### 4.1 Multi-batch alignments—all-by-all

Our primary use case for *Eclipse* is for combining multiple datasets as part of our processing workflow, reporting only features that are found in all datasets and that form a clique. Four human plasma datasets (*DS1*, *DS2*, *DS3*, *DS4*) were aligned, running in 16 s (Supplementary Code S1). *Eclipse* identified 3861 features (29% of the smallest dataset, *DS2*) and correctly matched 95% (362 of 381) of overlapping annotated features. In addition to the overlapping annotations, there were 111 nonoverlapping annotations which were used to measure alignment specificity. Most nonoverlapping annotations were a result of missed integrations during feature extraction. *Eclipse* generated three spurious matches, i.e. rows where annotations were mismatched (including both overlapping and nonoverlapping annotations). This is summarized in Supplementary Table S3.

Clustering settings can be relaxed if a user wishes to capture more features. We set the *diameter* to 2 and *minimum clique size* to 3. Compared to the strict “clique mode,” this matched 4530 (+669, 34%) features and 96% (+5, 367 of 381) of overlapping annotated features (Supplementary Code S2). There were 13 (+10) spurious matches. A visual representation of scaling results *DS1→DS2* is explained in Supplementary Figs S5 and S6, and all Plasma subalignments can be viewed in Supplementary Report S1.

### 4.2 Five disparate-matrix datasets—one-by-all

*Eclipse* is also used to identify equivalent features across biospecimens of different origins, like tissue types or biological fluids, relative to a reference. To demonstrate, rat plasma (*DS5*) was aligned to rat gastrocnemius (*DS6*), rat liver (*DS7*), rat heart (*DS8*); and rat white adipose (*DS9*). We performed a One-By-All alignment (Supplementary Code S3), in which *DS5* was aligned with all others, but *DS6–DS9* were not aligned to each other. This generates hub-spoke type clusters (Supplementary Fig. S7) which are captured by setting *minimum clique size* and *minimum group size* to 2 and *diameter* to 2. Intensity was disabled as well. The alignment finished in 10 s. Of the 12 461 features in *DS5*, 1600 had matches in all datasets, 3681 had partial matches, and 7180 did not have a match in any other dataset. Out of the 140 overlapping features that were present in all datasets, 130 were fully matched and the remaining 10 were partially matched.

### 4.3 Robust, recurring features—all-by-all

One potential use case for *Eclipse* is to better understand the robustness of feature detection across datasets over time, e.g. features in plasma datasets from different human cohorts, acquired months or years apart. For this, six plasma datasets (*DS1–4*, *DS10*, *DS11*), collected over 6 years on three different instruments were aligned, allowing for groups of size one (*minimum group size* and *minimum clique size* to 1), but still enforcing the requirement of groups being cliques (*diameter* set to 1) (Supplementary Code S4). This ran in 52 s. There were 1435 common features found among all datasets. 6376 features were found in *n ≥ 4* datasets (i.e. >50% of datasets). 47 522 clusters of size *n < 4* were formed, which are likely fragmented groups left by the strict clique-based clustering. Subalignment scaling and matching results can be viewed in Supplementary Report S1.

A similar experiment was conducted using all eleven datasets, with Intensity disabled and allowing for nonclique clustering (*diameter* set to 2). This revealed 976 features found in all datasets, and a total of 5334 features found in *n ≥ 6* datasets. All results, including *diameter* set to 1, 2, and 3, can be seen in Supplementary Table S2. We expect that features found in many datasets are real and robust, and high priority targets for identification. The scaling and matching reports for all 110 subalignments can be viewed in Supplementary Report S2.

### 4.4 Comparison to other tools

The most similar tools to *Eclipse* are M2S (Climaco Pinto *et al.* 2022), written in Matlab and *metabCombiner* (Habra *et al.* 2021, 2024), written in R. *metabCombiner* is capable of aligning *n > 2* datasets using a step-wise approach, which differs from *Eclipse’*s graph based approach. We aligned *DS1–4* using *metabCombiner* in *intersection* mode (Supplementary Code S5 and S6). Compared to *Eclipse’*s “clique mode” (*diameter *= 1), *metabCombiner* identified the same number of overlapping annotations (362 of 381), but a yielded a much higher number of spurious hits, 26 versus *Eclipse’*s three. We also observed that the results were dependent on dataset order. The annotation-evaluation results, with the six-plasma datasets and alternate parameters, can be viewed in Supplementary Tables S3 and S4. We also ran plasma and all datasets in both *union* and *intersection* mode, and the feature counts are reported on Supplementary Table S2.

An interesting feature of metabCombiner is its robust handling of various column gradients, a use case distinct from *Eclipse’*s original requirements. For this we have implemented a “prescaling” option, where a user may provide known descriptor values, such as the retention times of reference metabolites, to correct for large retention time differences prior to *Eclipse’*s data driven scaling. We are also open to implementing alternate or customizable scaling algorithms if superior approaches are demonstrated.

### 4.5 Benefits of Eclipse’s graph-based approach

*Eclipse’*s graph-based approach decouples the matching steps from the aggregation steps, which carries several advantages. For one, results are not dependent on insertion order. Second, feature matching can be visualized. As an example, annotation *Sphingosine* was not aligned by either *Eclipse* or *metabCombiner*. Using *Eclipse’*s *explain* method, we can visualize the component and observe that it was aligned in all datasets except *DS2*<->*DS4* (Supplementary Fig. S8). A final advantage is that we may re-cluster with new result params (*minimum clique size*, *minimum group size*, *diameter*) without rerunning the subalignments.

## 5 Conclusion

We offer *Eclipse* as a means to combine *n≥2* datasets, especially if a workflow requires symmetrical results (independent of order) and a Python environment. *Eclipse* is critical to our workflow and we intend to support it indefinitely. *Eclipse* is open source, and we welcome feedback and new feature requests from the metabolomics community. The code, instructions, and examples can be found as part of a larger processing toolset used by the Metabolomics Platform at the Broad Institute (including *Gravity—*feature clustering by RT/correlation, and *Blueshift—*drift correction) at https://github.com/broadinstitute/bmxp.

## Supplementary Material

btaf290_Supplementary_Data

## Data Availability

The data underlying this article are available in the article and in its online supplementary material.

## References

[btaf290-B1] Brunius C , Shi L, Landberg R. Large-scale untargeted LC-MS metabolomics data correction using between-batch feature alignment and cluster-based within-batch signal intensity drift correction. Metabolomics 2016;12:173. 27746707 10.1007/s11306-016-1124-4 PMC5031781

[btaf290-B2] Chen Z-Z , Pacheco JA, Gao Y et al Nontargeted and targeted metabolomic profiling reveals novel metabolite biomarkers of incident diabetes in African Americans. Diabetes 2022;71:2426–37. 35998269 10.2337/db22-0033 PMC9630088

[btaf290-B3] Climaco Pinto R , Karaman I, Lewis MR et al Finding correspondence between metabolomic features in untargeted liquid chromatography–mass spectrometry metabolomics datasets. Anal Chem 2022;94:5493–503. 35360896 10.1021/acs.analchem.1c03592 PMC9008693

[btaf290-B4] Clish CB. Metabolomics: an emerging but powerful tool for precision medicine. Cold Spring Harb Mol Case Stud 2015;1:a000588. 27148576 10.1101/mcs.a000588 PMC4850886

[btaf290-B5] Habra H , Kachman M, Bullock K et al metabCombiner: paired untargeted LC-HRMS metabolomics feature matching and concatenation of disparately acquired data sets. Anal Chem 2021;93:5028–36. 33724799 10.1021/acs.analchem.0c03693 PMC9906987

[btaf290-B6] Habra H , Meijer JL, Shen T et al metabCombiner 2.0: disparate multi-dataset feature alignment for LC-MS metabolomics. Metabolites 2024;14:125. 38393017 10.3390/metabo14020125 PMC10891690

[btaf290-B7] Koch S , Bueschl C, Doppler M et al MetMatch: a semi-automated software tool for the comparison and alignment of LC-HRMS data from different metabolomics experiments. Metabolites 2016;**6**:39. 10.3390/metabo6040039 PMC5192445 27827849

[btaf290-B8] Mak TD , Goudarzi M, Laiakis EC et al Disparate metabolomics data reassembler: a novel algorithm for agglomerating incongruent LC-MS metabolomics datasets. Anal Chem 2020;92:5231–9. 32118408 10.1021/acs.analchem.9b05763 PMC10926180

[btaf290-B9] Mascanfroni ID , Takenaka MC, Yeste A et al Metabolic control of type 1 regulatory T cell differentiation by AHR and HIF1-α. Nat Med 2015;21:638–46. 26005855 10.1038/nm.3868 PMC4476246

[btaf290-B10] Pluskal T , Castillo S, Villar-Briones A et al MZmine 2: modular framework for processing, visualizing, and analyzing mass spectrometry-based molecular profile data. BMC Bioinformatics 2010;11:395. 20650010 10.1186/1471-2105-11-395 PMC2918584

[btaf290-B11] Smith CA , Want EJ, O'Maille G et al XCMS: processing mass spectrometry data for metabolite profiling using nonlinear peak alignment, matching, and identification. Anal Chem 2006;78:779–87. 16448051 10.1021/ac051437y

[btaf290-B12] Smith R , Ventura D, Prince JT. LC-MS alignment in theory and practice: a comprehensive algorithmic review. Brief Bioinform 2015;16:104–17. 24273217 10.1093/bib/bbt080

[btaf290-B13] Tahir UA , Katz DH, Avila-Pachecho J et al; NHLBI Trans-Omics for Precision Medicine 1 Consortium. Whole genome association study of the plasma metabolome identifies metabolites linked to cardiometabolic disease in black individuals. Nat Commun 2022;13:4923. 35995766 10.1038/s41467-022-32275-3 PMC9395431

[btaf290-B14] Vatanen T , Jabbar KS, Ruohtula T et al Mobile genetic elements from the maternal microbiome shape infant gut microbial assembly and metabolism. Cell 2022;185:4921–36.e15. 36563663 10.1016/j.cell.2022.11.023 PMC9869402

